# Alveolar type 2 epithelial cell senescence and radiation-induced pulmonary fibrosis

**DOI:** 10.3389/fcell.2022.999600

**Published:** 2022-11-02

**Authors:** Shenghui Zhou, Jiaojiao Zhu, Ping-Kun Zhou, Yongqing Gu

**Affiliations:** ^1^ Hengyang Medical College, University of South China, Hengyang, China; ^2^ Beijing Key Laboratory for Radiobiology, Beijing Institute of Radiation Medicine, AMMS, Beijing, China

**Keywords:** radiation-induced pulmonary fibrosis, type 2 alveolar epithelial cells, cellular senescence, senescence associated secretory phenotype, therapy

## Abstract

Radiation-induced pulmonary fibrosis (RIPF) is a chronic and progressive respiratory tract disease characterized by collagen deposition. The pathogenesis of RIPF is still unclear. Type 2 alveolar epithelial cells (AT2), the essential cells that maintain the structure and function of lung tissue, are crucial for developing pulmonary fibrosis. Recent studies indicate the critical role of AT2 cell senescence during the onset and progression of RIPF. In addition, clearance of senescent AT2 cells and treatment with senolytic drugs efficiently improve lung function and radiation-induced pulmonary fibrosis symptoms. These findings indicate that AT2 cell senescence has the potential to contribute significantly to the innovative treatment of fibrotic lung disorders. This review summarizes the current knowledge from basic and clinical research about the mechanism and functions of AT2 cell senescence in RIPF and points to the prospects for clinical treatment by targeting senescent AT2 cells.

## 1 Introduction

Radiation-induced pulmonary fibrosis (RIPF) is the most common complication in patients with thoracic tumor radiotherapy (Turrisi et al., 1999; Chang et al., 2015). Previous studies suggest that lung fibrosis (PF) is caused by chronic inflammation (Johnston et al., 1997). However, its pathogenesis has not yet been fully elucidated. Lung fibrosis can damage lung tissue and produce fibers, eventually causing fibrotic scarring (Noble et al., 2012). The predominant pathological characteristics of RIPF include abnormal interstitial inflammation and fibrosis, loss of function of alveolar epithelial cells, and activation of mesenchymal cells. These characteristics destroy the structure of lung tissue, cause pulmonary fibrosis, respiratory failure, and ultimately death (Marks et al., 2003; Tsoutsou and Koukourakis 2006; Graves et al., 2010; Marks et al., 2010; Zanoni et al., 2019). Lung transplantation was the only plausible approach to treating RIPF until the advent of antifibrotic therapy (Ebert et al., 2015). Alveolar type 2 epithelial cells (AT2) are the main target cells that respond to ionizing radiation (IR) (Desai et al., 2014). They secrete surface-active substances and participate in the immune response and stem cell differentiation (Tesei et al., 2009; Beers and Moodley 2017). They also play an important role in lung development and injury recovery.

Cellular senescence is a state in which the cell cycle is at an irreversible standstill (Ogryzko et al., 1996; Wada et al., 2004; Herranz and Gil 2018). Cellular senescence could increase the expression of a senescence-associated secretory phenotype (SASP), which is involved in cell proliferation, inflammation, and induction of epithelial-mesenchymal transition (EMT) and promotion of fibrosis (Kaarniranta et al., 2022). Progressive telomere attrition, DNA damage, reactive oxygen species (ROS), and metabolism are considered the leading causes of cellular senescence. IR can also induce these changes (Araya et al., 2019).

**FIGURE 1:**
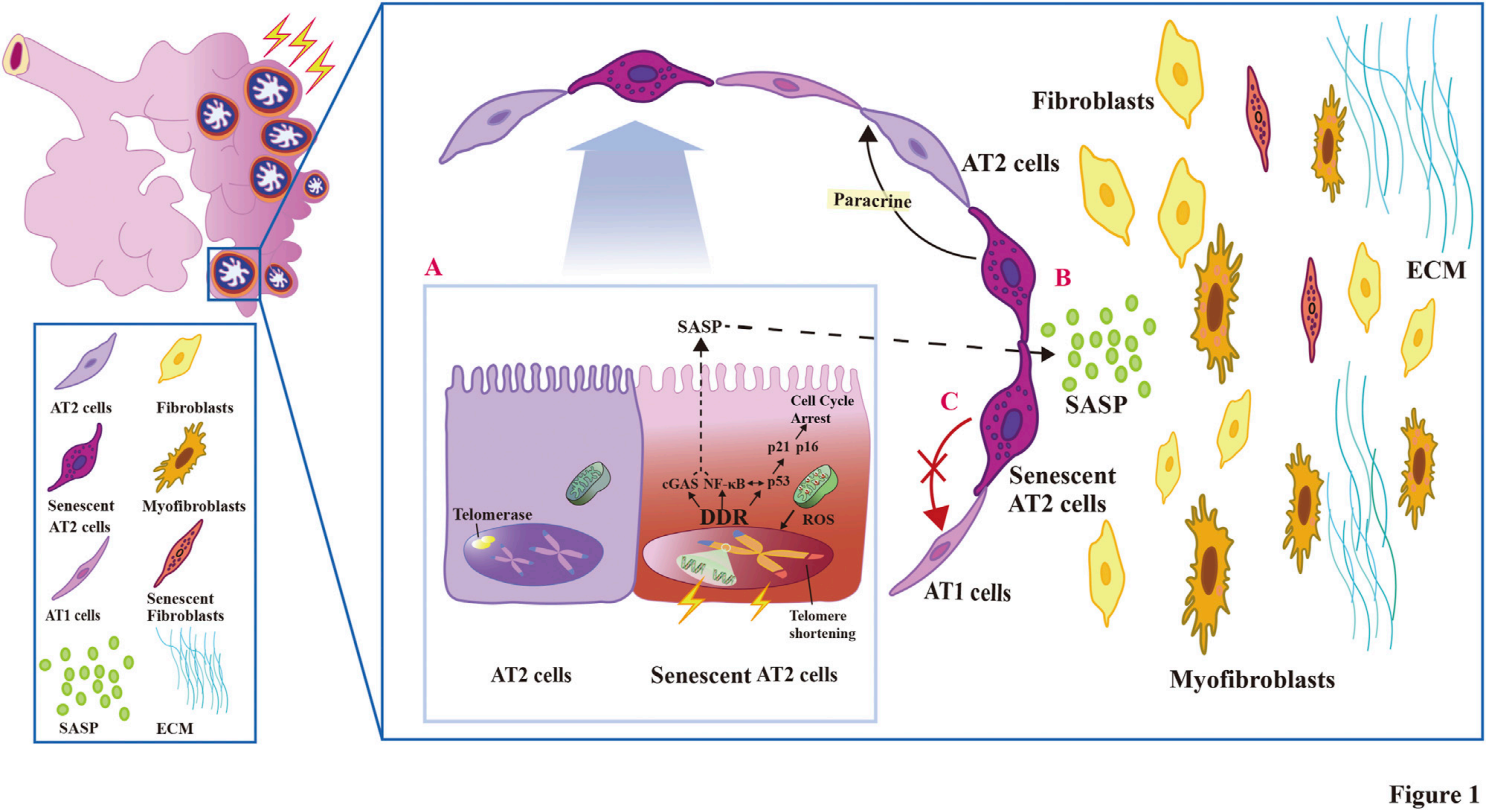
The relationship and pathogenesis associated with AT2 cell senescence and the development of fibrosis in RIPF.

This review summarizes the current knowledge about the mechanism and roles of AT2 cell senescence in RIPF (Figure 1). Recent publications on effective therapeutic strategies targeting senescent AT2 cells in RIPF are also generalized.

## 2 Biological role and the pathological response of alveolar epithelial type 2 cell

### 2.1 AT2 cells act as “tissue stem cells” in the normal lung tissue

In normal lung tissue, regional stem and progenitor cells contribute to maintaining the balance of lung homeostasis and repair (Hayes et al., 2011; Alysandratos et al., 2021). In fact, alveolar epithelial cells are divided into alveolar type 1 epithelial (AT1) and AT2 cells; the AT2 cells act as “tissue stem cells”. Lineage tracing experiment showed that AT2 cells are defined by their expressions of surfactant protein C (SFTPC). These cells have a longer-term capacity for self-renewal and the potential to differentiate into AT1 cells, which are mature tissue stem cells (Liu Q. et al., 2019). The analysis suggests that AT2 cells showed greater clonogenic potential and functional heterogeneity *in vivo*. The AT2 cells, which can differentiate into AT1, are only a subset of the AT2 cells (Rackley and Stripp 2012; Sun et al., 2021). The functional heterogeneity of AT2 cells could be closely related to the microenvironment in lung tissues; for example, WNT signaling leads to microenvironmental changes in the lung that allow AT2 cells to perform stem cell functions; as in the microenvironment of lung fibrosis, various types of AT2 cells perform different functions (Stegemann-Koniszewski et al., 2016; Nabhan et al., 2018; Yao et al., 2019). AT2 cells are the source of alveolar surfactant (Griese 1999). AT2 cells are the alveolar defense wall that inhibits microbial growth by secreting surface-active substances and recruiting effector immune cells and secreting antimicrobial peptides (Hiemstra et al., 2015). AT2 cells are also responsible for regenerating and replenishing lung epithelial cells. Consequently, AT2 cells have the potential to contribute to repair following a lung injury.

### 2.2 Pathological response of the AT2 cell in RIPF

AT2 cells are the critical target cells for lung injury caused by IR. Their dysfunction is a vital effector process of lung injury (De Ruysscher et al., 2019). In response to IR induction, AT2 cells suffer apoptosis due to DNA damage. The onset of AT2 apoptosis leads to stem cell depletion, failure of structural repair of lung tissue, and is a precursor to fibrosis formation (Liang et al., 2016). Under IR stimulation, AT1 cells lose the ability to maintain normal alveolar structure and gas exchange. After the initial direct and indirect radiation damage, the damage leads to the production of reactive oxygen species (ROS) (Khan et al., 2003). ROS can release damage-associated molecular patterns (DAMP) through AT2 cells and vascular endothelial cell injury, recruiting inflammatory cells such as monocytes and neutrophils that have pattern recognition receptors on their surfaces (Feldman et al., 2015). Numerous cytokines and chemokines from AT2 cells and other lung cells are active in irradiated lung tissue and have been implicated as drivers of RIPF, such as TGF-β activity stimulates fibroblast differentiation to myofibroblasts (Rube et al., 2000). Endoplasmic reticulum (ER) stress and mitochondrial dysfunction following IR stimulation are also factors in developing functional abnormalities in AT2 and triggering fibrosis. Our previous studies documented that the epithelial-mesenchymal transition (EMT) of AT2 cells upon irradiation plays an important role in the development of RIPF (Liu Z. et al., 2019; Liang et al., 2021; Wang et al., 2021; Yan et al., 2022). Our research supports an idea that AT2 cells produce early effects after irradiation and acts as an initiating event in the pathogenesis of RIPF. AT2 cells are also involved in crucial immune responses (Bissonnette et al., 2020). Furthermore, AT2 cells transport immunoglobulins and produce components of the complement system (Fehrenbach 2001; Datta et al., 2020). It has been shown that when AT2 cells are subjected to chronic and persistent microstimulation, PF can be triggered (Kinoshita and Goto 2019). In many investigations of PF, it is suggested that a decrease in the number of AT2 cells, as well as a targeted stimulation of AT2 cells to make them impaired, promotes PF development (Sisson et al., 2010). Many studies indicate AT2 cell senescence as a persistently stained form of IR-induced injury (Hernandez-Segura et al., 2018; Baselet et al., 2019). Furthermore, senescent AT2 cells lose the ability to proliferate and differentiate into AT1 cells due to their stromal epithelial transformation effect. They transform into fibroblasts (Naikawadi et al., 2016; Chen et al., 2019), preventing effective reconstruction of the structure of lung tissue. The injury gradually aggravates with IR stimulation, leading to lung function disorder and induction of RIPF. Substantial evidence suggests that the loss and dysfunction of AT2 cells play a central role in fiber proliferation (Parimon et al., 2020). Thus, AT2 cells serve as drivers of lung fibrosis in RIPF.

## 3 The pathogenesis role of AT2 cell senescence in RIPF

### 3.1 Senescence of the AT2 cell is a critical driver event of RIPF

RIPF is believed to be the end point of a continuous progression of radiation-induced lung injury (RILI). Electron microscopy showed damaged lung epithelial cells, thickened secretions of goblet cells mucus, swelling of the basement membrane, and changes in endothelial cells (Hanania et al., 2019). When the alveolar epithelium is continuously damaged, AT2 cells undergo distinctive biochemical and morphological changes and become dysfunctional and senescent. These processes are followed by senescence and activation of fibroblasts through paracrine effects, the initial pathological state of RIPF (Okunieff and Vidyasagar 2013; Chung et al., 2021). Activated fibroblasts differentiate into myofibroblasts, which exhibit contractile function and produce ECM components essential for the formation of PF (Chanda et al., 2019). In isolated lung tissues of RIPF patients, the expression of p16^INK4a^ and p21^CIP1^ increased in AT2 cells, indicating a positive correlation between the degree of senescence of AT2 cells and the progression of pulmonary fibrosis disease (Beausejour 2003; Soysouvanh et al., 2020; Jiang et al., 2017). So far, studies in mouse models with a bleomycin-induced fibrosis-like phenotype (a chemotherapeutic drug) support the hypothesis that senescence contributes to fibrosis (Li et al., 2021). Growing evidence suggests that cell senescence is a causative factor in RIPF (He et al., 2019). Therefore, cellular senescence is a critical physiological process that should be targeted to explore an effective treatment for RIPF.

Cellular senescence was first described more than 50 years ago followed the Hayflick Limit’ concept. This concept observed that normal human fibroblasts underwent a finite number of divisions before ceasing to proliferate when grown in the culture (Macieira-Coelho 1995; Shay and Wright 2000). Cellular senescence is a complex process and is usually accompanied by the activation of the DNA damage response (DDR) after IR. DDR is operated by activating the tumor suppressor protein p53 and upregulating the cyclin-dependent kinase inhibitors (CDKIs) p21^CIP1^ and p16^INK4a^ (Huang and Zhou 2021; Shmulevich and Krizhanovsky 2021). Furthermore, unlike proliferating cells, senescent cells are normally characterized by changes in cell size and morphology and exhibit senescence-associated heterochromatin foci formation, lipofuscin deposition, DNA damage foci, and Lamin B1 deletion. Senescence causes the secretion of high levels of factors, including growth factors, cytokines, chemokines, and proteases, known as the senescence-associated secretory phenotype (SASP).

AT2 cells are stem cells that are extraordinarily sensitive to senescence, and AT2 cell senescence leads to stem cell exhaustion and failure of repair mechanisms (Barnes et al., 2019). Compared to young AT2 cells, old AT2 cells showed decreased expression of the AT2 cell marker Surfactant Protein C along with increased expression of the AT1 cell marker Hopx, accompanied by increased WNT/β-catenin activity. These results suggest that chronic WNT/β-catenin activity in PF contributes to increased AT2 cell senescence and reprogramming (Lehmann et al., 2020). Dysfunctional alveolar epithelial cells are associated with senescence and play a key role in the remodeling process of abnormal lung injury. AT2 cell senescence is a prominent pathological manifestation of RIPF (Morgan et al., 1995; Schafer et al., 2017; Habermann et al., 2020). AT2 cell senescence can cause tissue repair, regeneration failure, and loss of alveolar epithelial integrity (Hernandez-Gonzalez et al., 2021). Furthermore, AT2 cell senescence probably affects cell-to-cell communication with fibroblasts and immune cells and are more susceptible to alterations in senescence such as telomere shortening and mitochondrial dysfunction compared to normal cells (Alder et al., 2015; Bueno et al., 2015). However, in myofibroblasts, telomere shortening does not occur (Álvarez et al., 2017). Single-cell RNA sequencing research indicates that senescent AT2 cells increase in lung tissue from PF patients compared to normal human lung tissue and that they can activate myofibroblasts through multiple pathways (Reyfman et al., 2019).

Senescent lung fibroblasts can induce myofibroblast differentiation in a paracrine manner, suggesting that they express profibrotic protein. In mouse model of pulmonary fibrosis treated with bleomycin, persistent fibrosis is attributed to the aggregation of senescent and antiapoptotic myofibroblasts (Hecker et al., 2014). Alveolar macrophages are cells that inhabit the lung for the long term. Their numbers and changes associated with senescence can be regulated by changes associated with the alveolar microenvironment, independent of circulating signals. Normal macrophages originally functioned to remove harmful cells, but senescent macrophages cannot clear aggregated senescent cells. In the microenvironment of persistent immune senescence, it facilitates the transition to a profibrotic state. Likewise, aberrant signals from senescent inflammatory cells and fibroblasts may activate the expression of genes associated with inflammation and fibrosis in epithelial cells of PF (Chakraborty et al., 2022). In summary, the occurrence of cell damage and senescence in the lung contributes to pulmonary fibrosis, but the senescence of alveolar epithelial cells may be a central driver in RIPF rather than the senescence of other cells.

### 3.2 SASP offers a scaffold for the senescence of AT2 cells

Senescent AT2 cells secrete several inflammatory proteins called SASP (senescent-associated secretory phenotype). SASP comprises a series of cytokines such as pro-inflammatory cytokines (IL-6, IL-1α, IL-18, etc.), growth factors (TGF-β), chemokines (CXCL10, CXCL12), and matrix remodeling enzymes (MMPs, matrix metalloproteinases). Furthermore, they serve as a new mechanism for the cellular senescence effect (Coppé et al., 2008; Freund et al., 2010; Aird et al., 2016; Yin et al., 2021). Senescence is an inflammatory state in AT2 cells. These proteins cause low-grade chronic inflammation and diseases in the organization and accelerate the aging process of senescent AT2 cells and their neighboring cells through paracrine action (Evangelou et al., 2022). SASP develops gradually, triggering chronic inflammation and loss of tissue function, culminating in AT2 cell senescence and lung fibrosis (Kuilman et al., 2010; Kang and Elledge 2016; Liu et al., 2021). Significantly, senescent AT2 cell clearance reduces the expression of SASP factors such as IL-6, TGF-β, and MMP12, which play a decisive role in the regulation of pulmonary fibrosis and lung function (Jeon et al., 2017; Schafer et al., 2017; Prata et al., 2018). Thus, SASP is an essential mediator of AT2 cells in the pathology of pulmonary fibrosis.

Recently, sufficient evidence suggests that extracellular vesicles (EVs) secreted from senescent AT2 cells modulate the phenotype of recipient cells, for example, by accelerating senescence. This process results in inflammation and stem cell (such as AT2 cells) dysfunction, similar to SASP factors (Raposo et al., 1996; Fujita et al., 2015). EVs transport proteins secreted explicitly from senescent cells to promote the occurrence of fibrosis in RIPF (Fujita et al., 2016; Takahashi et al., 2017; Kadota et al., 2020). In particular, TGF-β, a component of SASP with enormously high content, is a crucial promoter of EMT in AT2 cells (Zhou et al., 2012). It promotes mesenchymal cell protein expression and subsequent EMT, allowing for further pulmonary fibrosis (Xu et al., 2009; Bai et al., 2017; Lee et al., 2021). In summary, SASP is a marker of AT2 cell senescence. It acts with other cells in the lung through paracrine action and other factors to induce widespread cellular senescence, promoting pulmonary fibrosis.

### 3.3 Activation of fibroblasts and alteration of the extracellular matrix induced by AT2 senescence

Remodelling after lung tissue injury is critical for normal lung homeostasis and function (Vaughan et al., 2015). Senescent AT2 cells express SASP, inducing massive proliferation and activation of lung fibroblasts and myofibroblasts to repair the injury. When the repair is out of control, the basement membrane (BM) disrupts, and the extracellular matrix (ECM) is excessively produced, ultimately leading to RIPF (Demaria et al., 2014; De Biasi et al., 2020; Zhang et al., 2020; Marquez-Exposito et al., 2021; Zhang Y. et al., 2022). The ECM was initially thought to be a simple scaffold that supported the anatomy of the lung, providing structural support to the airways. However, ECM components were subsequently found to respond strictly to changes in the functional signals of its surrounding cells, like AT2 cells (Theocharis et al., 2016). ECM includes collagen, hyaluronic acid, connexin, and other components. It is integral to normal tissue healing and pathological processes (Rowley et al., 2019). When IR causes AT2 cell senescence, SASP can promote the EMT of AT2 cells to generate fibroblasts and myofibroblasts; On the other hand, SASP activates and multiplies fibroblasts; SASP also acts on myofibroblasts to induce senescence and aggravate inflammatory responses; these lead to excessive proliferation and abnormal activation of myofibroblasts, producing a large number of components of the ECM such as collagen and finally inducing RIPF (Laberge et al., 2012; Xu et al., 2015; Haydont et al., 2019; Jin et al., 2020).

## 4 Regulatory pathways in radiation-induced AT2 cell senescence

### 4.1 Telomeres and telomerase linked to AT2 cell senescence

Previous studies found that telomere shortening results in AT2 cell senescence. Telomere shortening after each mitosis was observed as early as 1960 (Hayflick and Moorhead 1961). However, they trigger sustained DDR if they are too short. This phenomenon is true for some nonfunctional telomeres, where DDR recognizes DNA damage and repairs it to arrest the cell cycle (Allsopp et al., 1992). Studies have shown that senescent AT2 cells can have cell cycle arrest due to the shortening of the telomere (Fernandez et al., 2022). The telomerase complex protects the telomeres from DNA damage and maintains the telomere intact. (Schratz et al., 2021). The minimum length ensures the successful binding of telomere protection proteins to telomeres (Li et al., 2017). If the protective protein or telomerase activity is altered, telomere uncapping causes DNA damage and poor repair, triggering AT2 cell senescence (Zhang et al., 2016).

Studies on AT2 cells from patients with pulmonary fibrosis have found a shorter telomeric length (Duckworth et al., 2021). IR is a critical pathological factor that contributes to telomere attrition (Aubert and Lansdorp 2008). On one hand, IR causes a sustained DDR; on the other hand, it leads to telomeric damage and dysfunction, both of which can induce AT2 cell senescence. In TRF1-deficient AT2 cells, activation of the DNA damage response exacerbates pulmonary fibrosis (Hsu et al., 2007). In radiation-induced lung injury (RILI), increased cell division during radiation-induced pneumonia increases telomeric wear and tear. Telomeres are susceptible to oxidative stress induced by chronic inflammation and IR, thus accelerating AT2 cell senescence (von Zglinicki, 2002; Wu et al., 2019; Huang and Zhou 2020; Wang et al., 2020; Wang et al., 2020). Furthermore, IR promotes AT2 cell senescence by inducing TPP1 ubiquitination and degradation that uncaps the telomeres (Zemp and Lingner 2014; Smith et al., 2020). Telomere dysfunction stimulates sustained DDR signaling, vital for AT2 cell senescence and its phenotype. For example, sustained DDR signaling releases IL-6, a key cytokine of SASP (Ghosh et al., 2012).

In addition, telomerase regulates NF-κB, COX-2, and other pathways that enhance inflammatory effects. It also exacerbates cellular senescence through autocrine or paracrine secretions (Chung et al., 2006; Lagnado et al., 2021). Telomere-associated inflammation limits tissue regeneration capacity and accelerates the senescence process by impairing stem cell differentiation and division of AT2 cells (Alder et al., 2015). To conclude, telomeres and telomerase play a crucial role in AT2 cell senescence and, by extension, in RIPF exacerbated by cell senescence.

### 4.2 Cell cycle arrest in AT2 cell senescence

In RIPF, cell cycle arrest was observed in senescent AT2 cells. The mechanisms of AT2 cell senescence leading to RIPF are not fully elucidated. There seems to be a link between the tumor suppressor genes p53 and AT2 cell senescence. Once IR damages DNA, p53 is stably overexpressed by DDR signaling for DNA repair (Lowe et al., 1993; Pitolli et al., 2019). Under irreparable DNA damage, p53 induces cellular senescence, arresting the cell cycle (Engeland 2018). Furthermore, increased p53 activity in AT2 cells reduces their ability to proliferate and differentiate, thus promoting the progression of the senescent phenotype (Chen et al., 2020). Simultaneously, p53 also mediates the cell cycle-dependent kinase inhibitor p21^CIP1^, which causes cell cycle arrest and DNA damage-induced cellular senescence in the G1/S transition (Deng et al., 1995; Kim et al., 2017).

Many studies have shown that IR activates p53 and stabilizes its activity (Chen et al., 2021). Previous studies have detected persistently higher expression of p53 in IR-induced senescent AT2 cells (Mathew et al., 2011; Beach et al., 2018). RNA and protein levels of p53 were increased in a mouse model of pulmonary fibrosis, similar to the expression of RIPF in the lung tissue (Nam et al., 2021). However, when a senolytic drug was used to improve the state of pulmonary fibrosis and remove senescent AT2 cells in mice, the p53 content was visibly reduced (Yao et al., 2021). Additionally, the senescent phenotype of AT2 cells is alleviated when the p53-deficiency is present (Zhang et al., 2021). Fanconi anemia (FA) is extremely sensitive to oxidative stress (ROS), which is DNA damage, and p53 deletion can alleviate the symptoms of HSPCs. Activation of p53 at high ROS levels leads to increased cell cycle arrest, senescence, and apoptosis of HSPCs (Joenje et al., 1981; Abbas et al., 2010; Brosh et al., 2017). Analogically, p53 promotes IR-induced cellular senescence through FA-like regulatory effects since IR-induced DNA damage is predominantly mediated by ROS generation. In conclusion, in senescent AT2 cells, p53 expression was steadily increased, and cell cycle arrest exhibited a senescent phenotype.

p21^CIP1^ is a cell cycle regulator, a senescence-inducing factor, and a tumor suppressor (Morris-Hanon et al., 2017). It plays a pivotal role in apoptosis, differentiation, induction of pluripotent stem cells, DNA repair, transcription, and cell migration (Avelar et al., 2020). Overexpression of p21^CIP1^ induces cell differentiation in various normal and tumor cells, including AT2 cells (Cheng et al., 2000; Warfel and El-Deiry 2013). In the RIPF mouse model and senescent AT2 cells, the level of p21^CIP1^ increased significantly (Citrin et al., 2013; Yosef et al., 2017; Sainz de Aja et al., 2021). RIPF p21^CIP1^ and p16^INK4a^ levels were reduced by senolytic drug treatment (He et al., 2019). Furthermore, inhibition of the PI3K/Akt pathway removes p21^CIP1^ from the cytoplasm and attenuates RIPF (Beeser et al., 2005; Thillai et al., 2017). In conclusion, increased p21^CIP1^ expression affects the state of AT2 cells and induces senescence by regulating the cell cycle.

p16^INK4a^ is a senescence marker and induces AT2 cell senescence (He and Sharpless 2017; Zysman et al., 2020). Past studies found an association between the effect of reactive oxygen species (ROS) on p16^INK4a^ and the β-catenin/Wnt signaling pathway in senescence in AT2 cells (Lehmann et al., 2020). In lung tissue from RIPF patients and a mouse model of pulmonary fibrosis treated with bleomycin, the content of p16I^NK4a^ increased with senescent cells, suggesting the correlation between p16^INK4a^ levels and the severity of fibrosis (Liggett and Sidransky 1998; Carraro et al., 2020). In earlier studies, BubR1 was associated with p16^INK4a^ expression and senescence phenotype, which paved the way for future studies on the relationship between p16^INK4a^ and AT2 cells with stem cell functions. Excessive expression of p16^INK4a^ under various stimuli such as DDR, telomere erosion, and ROS by IR causes irreversible cell cycle arrest (Kang et al., 2015; Bernard et al., 2020).

### 4.3 Senescence of AT2 cells triggered by the cGAS-STING pathway

In many studies on cell senescence, new mechanisms and pathways have been revealed. The latest research reveals the physiological importance of this ancient, but non-canonical cGAS-STING pathway in damage-triggered AT2 cell senescence and fibrotic diseases in the lung (Zhang D. et al., 2022) cGMP-AMP synthase (cGAS) is a cytoplasmic DNA sensor of the trigger-type interferon pathway. It is a new immune signaling mechanism activated by binding to double-stranded DNA such as microbial and self-DNA (Sun et al., 2013). cGAS produces a second messenger, cGAMP, that binds and activates STING (STImulator of IFN genes) (Wu et al., 2013). STING activates IFN regulatory factor 3 (IRF3) and NF-κB, producing interferons and inflammation-associated cytokines (Kato et al., 2017). Interestingly, cGAS is associated with chromatin during mitosis, indicating its other probable role in the regulation of the cell cycle. The deletion of cGAS abolishes the expression of the SASP gene and other markers of cell senescence, such as p21^CIP1^ (Yang et al., 2017). Drug-dependent pro-senescence cGAS-STING signaling drives the production of inflammatory SASP components (Constanzo et al., 2021).

When IR induces lesions, damaged DNA accumulates abnormally and substantially in the cytoplasm containing cGAS, triggering cell cycle changes and causing cellular senescence. Moreover, the cytoplasmic chromatin fragment (CCF) colocalizes with cGAS to form bright light spots, which appear in several cases of primary cellular senescence. CCF activates the cGAS-STING pathway in cellular senescence (Dou et al., 2017). Thus, the cGAS-promoting function of pulmonary fibrosis is also a focus of future attention.

## 5 Treatment and prospects

### 5.1 Current treatments

Currently, complete curative treatment is not available for RIPF, and the use of anti-inflammatory drugs does not achieve satisfactory therapeutic results. There are no established standardized guidelines for the treatment of RIPF. In previous clinical treatment, glucocorticoids and other anti-inflammatory drugs were commonly used, but in patients with pulmonary fibrosis, treatment was not effective or even risked exacerbation (Abratt et al., 2004; Canestaro et al., 2016). Recently, the multi-kinase inhibitor Nintedanib, which targets vascular endothelial growth factors among other growth factors, could reduce the incidence of lung fibrosis, and preclinical results have been promising; however, phase II trials are ongoing (De Ruysscher et al., 2017). Pirfenidone is another novel drug that downregulates collagens and growth factors and is worthy of a larger, well-controlled trial (Simone et al., 2007). Corticosteroids cannot be given at a fixed dosage, while inhaled steroids have shown efficacy for RIPF (Henkenberens et al., 2016). Amifostine is a radioprotector that functions as a free radical scavenging agent and reduces the risk of RIPF. Angiotensin-converting enzyme inhibitors (ACE inhibitors) have a significant antifibrotic effect and prevent the deposition of collagen fibers (Ghosh et al., 2009). Currently, however, drug prevention and treatment for RIPF are extremely limited, and the promotion of targeted precision therapy, in particular, may help improve the quality of life of patients with RIPF.

### 5.2 Anti-senescence therapy of AT2 cells

**FIGURE 2:**
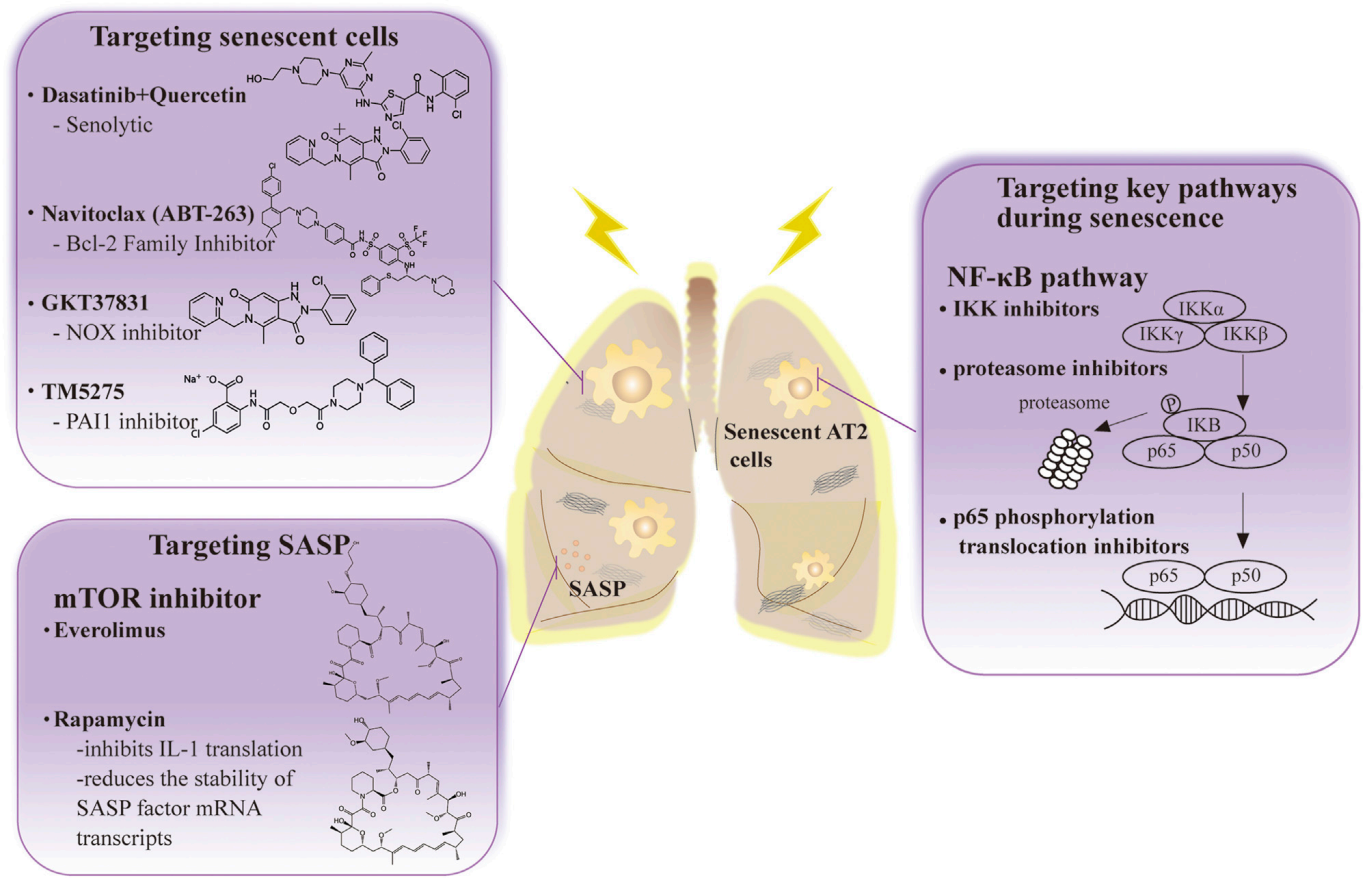
The anti-senescence therapy to prevent disease and extend healthy life span in RIPF.

Cellular senescence is one of the main etiologies of RIPF. Therefore, most investigators believe that cellular anti-senescence therapy may be successful in the future treatment of patients with RIPF (Lagoumtzi and Chondrogianni, 2021). This section summarizes current treatments for radiation-induced pulmonary fibrosis in the field of senescent cell-based therapy (Table 1) (Figure 2).

**TABLE 1:** Anti-senescence therapy with mechanism and effect in pulmonary fibrosis.

Treatment	Proposed mechanism(s)	Effect	References
Dasatinib + Quercetin	Senolytic (Bcl-xL inhibitor, ↓p16, ↓p21, ↑apoptosis)	↓Fibrosis in mice	Xu et al. (2018)
		↓Senescence cells	
Navitoclax (ABT-263)	↓Bcl-2	↓Fibrosis in mice	Pan et al. (2017)
		↓Senescence cells	
GKT37831	Antioxidant	↑Fibroblast apoptosis	Hecker et al. (2014)
		↑p53-dependent apoptosis	
		↓Replicative	
		↓Fibrosis in mice	
SkQ1	Antioxidant	↓Senescence biomarkers	Kolosova et al. (2012)
PAI1 inhibitor (TM5275)	↑p53, ↑apoptosis	↓Fibrosis in mice	Huang et al. (2012)
		Protected AT2 cells from senescence	Rana et al. (2020)
Metformin	↓NF-κB, ↓p16, ↓p21	↓SASP	Rangarajan et al. (2018)
	↓mTOR	↓Senescence cells	
Rapamycin	↓mTOR	↓SASP	Mullard (2018)
		↓Fibrosis in mice	Platé et al. (2020)
Everolimus	↓mTOR	↓SASP	Platé et al. (2020)

#### 5.2.1 Targeting senescent cells

Senolytic drug treatment is prominent in research on senescent cell apoptosis. These drugs have been used to treat many cancers (Childs et al., 2017). One is a combination of dasatinib (D), a complex kinase inhibitor, and quercetin (Q), a flavonoid and inhibitor. Treatment with D + Q is highly effective in reversing pulmonary fibrosis by negatively regulating p21^CIP1^ and p16^INK4a^ and promoting apoptosis in numerous mouse models of pulmonary fibrosis (Schafer et al., 2017; Xu et al., 2018). Depletion of senescent AT2 cells by senolytic drugs decreases fibrotic markers and increases epithelial cell marker expression (Lehmann et al., 2017). More interestingly, this treatment was beneficial to recovery in mice, and the effect was similar to the reduced fibrosis status observed in mice.

Clearance of senescent AT2 cells improves pulmonary fibrosis status in bleomycin-treated or IR-induced mouse models of pulmonary fibrosis. The number of AT2 cells that were senescent decreased and a trend towards reduced pulmonary fibrosis was observed after administering the ABT-263 drug navitoclax (Pan et al., 2017). It is an inhibitor of the BCL-2 family that removes senescent AT2 cells. Furthermore, it effectively decreases the viability of senescent human lung fibroblasts and murine embryonic fibroblasts (Lagares et al., 2017).

Oxidative stress is one of the factors that stimulate cellular senescence. Therefore, the use of antioxidant drugs is effective in cellular senescence and pulmonary fibrosis (Tse et al., 2008; Chang et al., 2016). Novel antioxidants such as the NADPH oxidase (NOX) inhibitor, GKT37831, inhibit lung fibrosis in aging mice, can cause injury to AT2 cells and fibroblast apoptosis, and interfere with the regulation of p53-dependent apoptosis and replicative senescence (Auten et al., 2009; Hecker et al., 2014; Sharma et al., 2014). At the same time, numerous studies have shown that mitochondria are involved in the process of oxidative stress. Hence, antioxidant drugs targeting mitochondria have extraordinary prospects in the treatment of pulmonary fibrosis. Sirtuin 1 (SIRT1) is an anti-senescence molecule involved in response to chronic inflammation and oxidative stress. Its activator SRT2104 protects against AT2 senescence in rats (Gu et al., 2020). SkQ1 is an antioxidant and reduces senescence biomarkers in aging mice (Kolosova et al., 2012). Furthermore, PAI-1 depletion blocks TGF-β1-induced senescence as well as a senescence-associated secretory phenotype in AT2 cells, and the PAI1 inhibitor (TM5275) blocked bleomycin-induced pulmonary fibrosis in mice and protected AT2 cells from further senescence by targeting the p53 pathway (Huang et al., 2012; Rana et al., 2020; Rana et al., 2020). These results suggest that targeting senescent AT2 cell clearance has significant therapeutic implications for pulmonary fibrosis.

#### 5.2.2 Targeting key pathways during senescence

P16^INK4a^ knockout mice had no physiological defects after growth, indicating that p16 is unnecessary for survival and organ development (Baker et al., 2011; Grosse et al., 2020) and their colleagues demonstrated that gene elimination in p16^INK4a^ positive senescent cells during accelerated and physiological aging causes age-related pathologies that extend the lifespan. Silencing p16 improves AT2 cell senescence and secretion of profibrotic mediators (Rana et al., 2020). Therefore, identifying inhibitors targeting senescence-associated pathways in AT2 cells may provide good ideas for the prevention and therapy of RIPF.

The NF-κB signaling pathway is also involved in IR-induced AT2 cell senescence. Targeted inhibition of its expression or activity will also effectively alleviate ROS and IR-induced inflammatory responses. Agents such as IKK inhibitors, proteasome inhibitors, and p65 phosphorylation translocation inhibitors effectively alleviate the inflammatory response (Pordanjani and Hosseinimehr 2016). However, due to crosstalk between NF-κB and p53, the application of targeted NF-κB pathway inhibitors requires more *in vitro* and *in vivo* experiments.

#### 5.2.3 Targeting SASP

A better therapeutic route for RIPF could involve the intervention of SASP expression. The genetic and pharmacological removal of senescent cells causes a simultaneous decrease in commonly described SASP factors, such as IL-6, TNF-α, IL-1α, and MCP-1, in tissues that show functional improvements in fibrosis pathologies (Wiegman et al., 2015). Inhibition of TGF-βalleviates lung fibrosis in AT2 cells (Yao et al., 2021). The mTOR pathway is essential in diseases associated with pulmonary fibrosis, and its inhibition prolongs the lifespan of animal models and reduces age-related pathological features (Liu and Sabatini 2020). Everolimus and Rapamycin are both mTOR inhibitors. However, the application of Everolimus in clinical studies has indicated a poor prognosis or even deterioration in patients (Mullard 2018). Rapamycin has recently been promising in reversing fibrosis in a mouse model of pulmonary fibrosis, primarily affecting ECM, cell metabolism, apoptosis, autophagy, and senescence (Platé et al., 2020). Rapamycin’s *in vivo* effects are attributed to its role as a SASP repressor since it inhibits IL-1 translation and reduces the stability of SASP factor mRNA transcripts (Wang et al., 2017). New applications are now possible for existing drugs. Metformin, which inhibits mTOR and prolongs lifespan in mice, inhibits and reverses fibrosis in the bleomycin mouse model and reduces fibrosis in PF (Rangarajan et al., 2018). Overall, targeting SASP could be a potential treatment for RIPF.

### 5.3 The advantage and limitations of anti-senescence therapy

According to the current patients status study, pulmonary fibrosis is a chronic and irreversible condition for which the only available treatment is symptomatic relief for collagen and inflammation (Hanania et al., 2019). However, early-stage of pulmonary fibrosis can sometimes be treated, and research on anti-senescence therapies for organ fibrosis is also focused on the prevention and treatment of fibrosis. The other benefit of anti-senescence therapy is targeted more precision therapy, specifically the removal or treatment of senescent cells, which may be less harmful to patients than current non-targeted treatments (Spagnolo et al., 2021). Although these anti-senescence drugs have shown excellent efficacy in animal studies, there is a lack of data to support their clinical application. Last but not least, anti-senescence therapy has a tremendous positive impact on the treatment of organ fibrosis in animal research at the experimental stage, but it still needs more clinical trials before it can be considered to have any significant effects (Parimon et al., 2021). However, there is a need for further studies, as currently, anti-senescence therapies can only target various senescence cells, making perfect, precise therapy impossible. As mentioned previously, the removal of p16^INK4a^ effectively mitigates the onset of senescence. Patients with pulmonary fibrosis cannot be treated using gene editing techniques such as gene knockouts, and these aspects greatly limit the application of targeted AT2-senescence therapy for RIPF. Therefore, more basic and clinical experiments are needed to demonstrate the feasibility of targeting aging AT2 cells for RIPF treatment.

## 6 Conclusion

Several reports indicate the pathogenic role of cellular senescence in the development of pulmonary fibrosis. Although AT2 cells play a crucial role in RIPF as effectors and stem cells of IR, they are the cells that undergo early manifestations of senescence after IR and can activate fibroblasts and alter the microenvironment in lung tissue, and their secreted SASP cause senescence of the immune environment in lung tissue and also affects other vital cells in the lung that occur in senescence. The new therapeutic strategy has shed the first light on RIPF patients. Cellular senescence, a pathogenic pathway, has innovative curative potential, and therapeutic approaches targeting cellular senescence will improve patient survival and quality of life shortly. Since AT2 cell senescence drives RIPF, embarking on anti-senescence therapy targeting AT2 cells in treatment may enable prevention or early intervention therapy for detecting RIPF after IR.

In addition, the lack of reliable evaluation metrics to assess the effectiveness of anti-senescence therapy in patients with pulmonary fibrosis is a serious challenge. Perhaps the application of senolytic drug therapy and old drugs that play new roles simultaneously and affect several pathways is a valuable option to overcome the treatment barriers of pulmonary fibrosis.
